# Bone lesion absorbed dose profiles in patients with metastatic prostate cancer treated with molecular radiotherapy

**DOI:** 10.1259/bjr.20170795

**Published:** 2018-01-31

**Authors:** Ana M Denis-Bacelar, Sarah J Chittenden, V Ralph McCready, Antigoni Divoli, David P Dearnaley, Joe M O’Sullivan, Bernadette Johnson, Glenn D Flux

**Affiliations:** 1 Chemical, Medical and Environmental Science Department, National Physical Laboratory, Teddington, UK; 2 Joint Department of Physics, The Institute of Cancer Research and The Royal Marsden Hospital NHS Foundation Trust, London, UK; 3 Department of Nuclear Medicine, Royal Sussex County Hospital, Brighton, UK; 4 Division of Radiotherapy and Imaging, The Institute of Cancer Research and The Royal Marsden Hospital NHS Foundation Trust, London, UK; 5 Centre for Cancer Research and Cell Biology, Queen’s University Belfast, Belfast, UK

## Abstract

**Objective:**

The aim of this study was to calculate the range of absorbed doses that could potentially be delivered by a variety of radiopharmaceuticals and typical fixed administered activities used for bone pain palliation in a cohort of patients with metastatic castration-resistant prostate cancer (mCRPC). The methodology for the extrapolation of the biodistribution, pharmacokinetics and absorbed doses from a given to an alternative radiopharmaceutical is presented.

**Methods:**

Sequential single photon emission CT images from 22 patients treated with 5 GBq of ^186^Re-HEDP were used to extrapolate the time–activity curves for various radiopharmaceuticals. Cumulated activity distributions for the delivered and extrapolated treatment plans were converted into absorbed dose distributions using the convolution dosimetry method. The lesion absorbed doses obtained for the different treatments were compared using the patient population distributions and cumulative dose–volume histograms.

**Results:**

The median lesion absorbed doses across the patient cohort ranged from 2.7 Gy (range: 0.6–11.8 Gy) for 1100 MBq of ^166^Ho-DOTMP to 21.8 Gy (range: 4.5–117.6 Gy) for 150 MBq of ^89^Sr-dichloride. ^32^P-Na_3_PO_4_, ^153^Sm-EDTMP, ^166^Ho-DOTMP, ^177^Lu-EDTMP and ^188^Re-HEDP would have delivered 41, 32, 85, 20 and 64% lower absorbed doses, for the typical administered activities as compared to ^186^Re-HEDP, respectively, whilst ^89^Sr-dichloride would have delivered 25% higher absorbed doses.

**Conclusion:**

For the patient cohort studied, a wide range of absorbed doses would have been delivered for typical administration protocols in mCRPC. The methodology presented has potential use for emerging theragnostic agents.

**Advances in knowledge:**

The same patient cohort can receive a range of lesion absorbed doses from typical molecular radiotherapy treatments for patients with metastatic prostate cancer, highlighting the need to establish absorbed dose response relationships and to treat patients according to absorbed dose instead of using fixed administered activities.

## Introduction

Prostate cancer is the most common cancer in males in the UK (2014), accounting for 26% of all new cancer diagnoses in males.^1^ Androgen deprivation therapy is the primary treatment for patients with metastatic prostate cancer, although the disease eventually progresses to the castration-resistant stage. Effective treatment is primarily palliative and disseminated bone metastases are often managed with molecular radiotherapy (MRT) towards the latter stages of the disease. A wide range of radiopharmaceuticals are available for bone pain palliation in patients with metastatic castration-resistant prostate cancer (mCRPC). These include bone-seeking calcium-analogues such as ^89^Sr-dichloride and ^223^Ra-dichloride and phosphates such as ^153^Sm-EDTMP, ^186^Re-HEDP and ^188^Re-HEDP.^2, 3^ More recently, newly emerging radiolabelled prostate-specific membrane antigen (PSMA)-binding radiopharmaceuticals show promise in treating bone and soft-tissue metastases.^4–6^

The efficacy of MRT relies on the delivery of high absorbed doses to the metastatic soft-tissue whilst minimizing the damage to healthy tissues. The red marrow is the major organ at risk for bone treatments and can, therefore limit the administered activity. Several models are available for dosimetry calculations in the normal skeleton,^7–10^ whilst lesion dosimetry is performed using sphere models or voxel dosimetry where the activity is assumed to be uniformly distributed through the volume. Accurate lesion dosimetry calculations require the determination of the activity distribution using sequential quantitative imaging. However, imaging of small bone metastatic lesions remains a challenge due to the limited spatial resolution of present clinical imaging systems and associated partial volume effects. A wide range of tumour absorbed doses are reported in the literature for MRT in mCRPC: 33 mGy MBq^–1^;^11^ 2.1 mGy MBq^^[Bibr b1]^^^–1^;^12^ and 3.7 mGy MBq^–1^;^13^ for ^186^Re-HEDP; 3.8 Gy MBq^–1^ for ^188^Re-HEDP;^14^ 37 mGy MBq^–1^ for ^89^Sr-dichloride;^15^ in excess of 62 mGy MBq^–1^ for ^131^I-MIP-1095 4.0 mGy MBq^–1^;^16^ and 4.4 mGy MBq^–1^;^17^ for ^153^Sm-EDTMP; 3.3 mGy MBq^–1^ for ^177^Lu-PSMA-DOTA-J591;^5^ and 13.1 mGy MBq^–1^ for ^177^Lu-DKFZ-PSMA-617.^18^ These radiopharmaceuticals exhibit different physiological effects and the absorbed doses reported have been calculated using different methodologies. These make comparisons within patients receiving the same treatment and between treatments challenging.

The aim of this study was to demonstrate the wide range of absorbed doses that can be delivered from typical administered activities of various MRT treatments used to treat mCRPC and to present the methodology to extrapolate the absorbed doses delivered from any chosen radiopharmaceutical to those that would be delivered if another radiopharmaceutical was administered. Patient-specific imaging from a cohort of patients treated with ^186^Re-HEDP were used to extrapolate the absorbed doses that would have been delivered if the same patients were treated with various treatments used for bone pain palliation.

## Methods and materials

### Clinical data

Available Phase II clinical trial data from a cohort of 22 patients treated with a median 5020 MBq of ^186^Re-HEDP and autologous peripheral blood stem cell transplantation were included. Sequential single photon emission CT (SPECT) imaging with up to five scans of the thorax and pelvis were acquired at approximately 1, 4, 24, 48 and 72 h following administration of the radiopharmaceutical. SPECT images comprised 128 × 128 voxels with a 4.67 mm^3^ voxel size. Further details of the patients and imaging acquisition and quantification can be found in.^13, 19,20^ All patients provided written consent to participate in the study, which was approved by the Royal Marsden NHS Foundation Trust and The Institute of Cancer Research Ethics Committee.

### Extrapolation of the time–activity curve

The ^186^Re-HEDP activity was quantified based on phantom experiments as described previously.^13^ Activities were extrapolated for other MRT treatments of bone metastases from prostate cancer, shown in Table 1: 450 MBq of ^32^P-Na_3_PO_4_(sodium orthophosphate),^21, 22^ 150 MBq of ^89^Sr-dichloride,^15, 23^ 37 MBq kg^–1^ of ^153^Sm-EDTMP (ethylenediamine tetramethylene phosphonate),^23^ 1100 MBq, of ^166^Ho-DOTMP (1,4,7,10-tetraazacyclododecane-1,4,7,10-tetramethylene-phosphonate),^24^ 37 MBq kg^–1^ of ^177^Lu-EDTMP^25, 26^ and 3300 MBq of ^188^Re-HEDP (hydroxyethylidene diphosphonate).^27, 28^ Information on the biological half-lives of ^153^Sm-EDTPM and ^177^Lu-EDTMP in bone lesions is not presently available. However, given that the uptake mechanism of bisphosphonates is comparable, the biological half-lives were assumed to be the same as that of ^186^Re-HEDP. A long biological half-life for ^32^P-Na_3_PO_4_ in bone lesions was assumed.^22^ Effective half-lives for ^188^Re-HEDP (bone lesions) and ^166^Ho-DOTMP (skeleton) were obtained from the literature.^14, 24^ Breen et al^15^ calculated a biological half-life for ^85^Sr in bone lesions of 50 days and assumed to be the same for ^89^Sr, whilst other sources suggest a value of 90 days. An average of these two values has been used in this study for ^89^Sr. Differences in bone uptake as a percentage of the administered activity for the different radiopharmaceuticals were also taken into account.^29^ PSMA-targeting radiopharmaceuticals were not considered in this study due to their different uptake mechanism as compared to ^186^Re-HEDP.

**Table 1.** List of radiopharmaceuticals, physical and effective half-lives, bone uptake and administered activity for typical molecular radiotherapy treatments of mCRPC

Radiopharm.	Physical half-life (d)	Effective half-life (d)	Bone uptake (%IA)	Administered activity (MBq)
^32^P-Na_3_PO_4_	14.268	13	20	450
^89^Sr-dichloride	50.563	29	65	150
^153^Sm-EDTMP	1.9379	1.6	70	37 (kg^–1^)
^166^Ho-DOTMP	1.1177	0.93	30	1100
^177^Lu-EDTMP	6.647	3.9	60	37 (kg^–1^)
^188^Re-HEDP	0.78500	0.66	30	3300

For any two given radiopharmaceuticals, the activity at any given time point *t* is calculated as:

$$A^{D}\left(t\right)=A_{0}^{D}exp\left(-\lambda_{eff}^{D}t\right)$$

$$A^{E}\left(t\right)=A_{0}^{E}exp\left(-\lambda_{eff}^{E}t\right)$$

where, in this study *D* and *E* refer to the delivered and extrapolated treatment plans, respectively. The effective decay constants ($\lambda_{eff}$) of the delivered and extrapolated treatment plans are defined as the sum of their respective biological ($\lambda_{bio}$) and physical ($\lambda_{phys}$) decay constants.

From the ratio of Equations (1) and (2) and solving for *A^E^*(*t*), the extrapolated activity at any given time point *t* is given by:

$$A^{E}\left(t\right)=\frac{A_{0}^{E}}{A_{0}^{D}}A^{D}\left(t\right)exp\left[\left(\lambda_{eff}^{D}-\lambda_{eff}^{E}\right)t\right]$$

Where *A*^*D*^(*t*) is the activity for the delivered radiopharmaceutical obtained from the quantified SPECT images, and $A_{0}^{D}$and $A_{0}^{E}$ are the administered activities for the delivered and extrapolated treatments plans respectively (Table 1). Equation 3 was used to extrapolate time–activity curves using available data on the biological retention and bone uptake for each radiopharmaceutical from the quantified SPECT images of ^186^Re-HEDP.

### Dosimetry

Following the Medical Internal Radiation Dosimetry pamphlet No. 17,^30^ the convolution dosimetry method was used, whereby the absorbed dose to any given target voxel (*voxel_t_*) is calculated by:

$$D_{voxel_{t}}=\sum_{s=1}^{N}{\tilde{A}}_{voxel_{s}}\times S\left(voxel_{t}\leftarrow voxel_{s}\right)$$

where ${\tilde{A}}_{voxel_{s}}$ is the voxelized cumulated activity distribution and $S\left(voxel_{t}\leftarrow voxel_{s}\right)$ is the absorbed dose S-value voxel kernel.

Voxelized cumulated activity distributions for ^186^Re-HEDP and the extrapolated treatments were obtained from the integration of the corresponding time–activity curves. Integration was performed between phases defined by the scan time points using an exponential or a trapezoidal fitting method subject to whether the activity between any two scan decreased or increased, respectively.

Absorbed dose voxel kernels for ^32^P, ^89^Sr, ^153^Sm, ^166^Ho, ^177^Lu, ^186^Re and ^188^Re were generated using the EGS ++ class of the general purpose Monte Carlo code EGSnrc v. 4.^31, 32^ These were calculated in a voxelized geometry in a soft-tissue density medium with dimensions of 21 × 21 × 21 voxels and the same voxel size as the imaging data, 4.67 mm. The nuclear decay data were obtained from the Medical Internal Radiation Dosimetry RADTABS program.^33^ The developed software code has been previously verified by comparison with available voxel S-values.^30, 34^

The individual bone lesions were outlined from the calculated absorbed dose distributions on a HERMES workstation (Hermes Medical Solutions, Stockholm, Sweden) as part of a previous study^13^ and saved in eXtensible Markup Language format for further analysis. The convolution dosimetry and post-processing data analyses were carried out using MATLAB 2016b (The MathWorks Inc., Natick, MA, US).

To reduce the effect of partial volume and the impact of the different spatial resolutions associated with each radionuclide, lesion peak absorbed doses were obtained by averaging the maximum voxel absorbed dose with the first nearest neighbour voxel values. Absorbed dose profiles were obtained for the delivered and extrapolated treatments, representing the distribution of bone lesion absorbed doses for the patient population. These were converted to cumulative dose volume histograms (cDVH) to study the spatial distribution of irradiated lesions across the patient cohort for each treatment. The patient population disease volume was defined as the sum of the metastatic tumour burdens for the 22 patients. The minimum absorbed dose to which 50% of the total volume was irradiated was calculated (D50).

## Results

**Figure 1.**
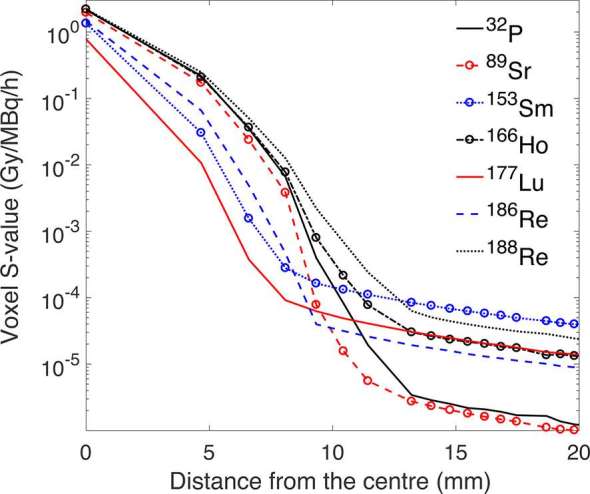
Absorbed dose voxel kernels as a function of the distance from the centre voxel for the radionuclides studied. Note the logarithmic scale of the vertical axis.

The dose voxel kernels for all the radionuclides studied are shown in Figure 1, and the S-values for the self-irradiation and the first nearest neighbour voxels are given in Table 2. The absorbed dose per decay rapidly decreases with the distance from the centre voxel, with a reduction of 89–99% in the first nearest voxel as compared to the self-irradiation voxel S-value. At distant voxels, higher S-values are observed for the radionuclides that also decay via γ-ray emission. The contribution to the lesion absorbed doses is negligible, as their contribution is four orders of magnitude lower.

**Table 2.** Voxel S-values for the self-irradiation and first nearest voxels for the radionuclides studied, where the number in the parentheses is the statistical uncertainty referred to the corresponding last digits of the S-value

Radionuclide	S-value (Gy MBq^–1^ ^–1^)	
Self-irradiation	Nearest neighbour
^32^P	2.1437 (5)	0.218209 (3)
^89^Sr	1.9573 (5)	0.1720 (2)
^153^Sm	1.3444 (4)	0.03031 (7)
^166^Ho	2.1929 (8)	0.2092 (3)
^177^Lu	0.7782 (3)	0.01066 (3)
^186^Re	1.4561 (5)	0.0655 (1)
^188^Re	2.2032 (8)	0.2477 (3)

**Figure 2.**
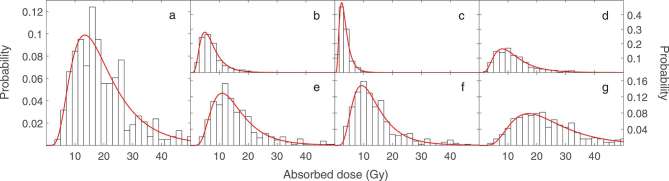
Absorbed dose profiles delivered by ^186^Re-HEDP (a) and and extrapolated for typical administered activities of ^186^Re-HEDP (b) ^177^Lu-EDTMP (c) ^32^P-Na_3_PO_4_ (d) ^153^Sm-EDTMP (e) ^166^Ho-DOTMP (f) and ^89^Sr-dichloride (g) obtained from the 379 bone lesions in the 22 patients. Note the three different ranges for the vertical axes: (a); (b-d); (e-g).

A total of 379 bone lesions were identified in 22 patients. The absorbed dose profiles fitted to log-normal distributions delivered by ^186^Re-HEDP and extrapolated for the MRT treatment plans in Table 1  are shown in Figure 2a and Figure 2b–g, respectively.

The median, minimum, maximum and D50-absorbed doses are shown in Table 3. A range of absorbeddoses could be delivered for typical empirically determined administered activities of the different treatments studied. If the same patient population was treated with ^89^Sr-dichloride, a 25% higher median lesion absorbed dose would have been delivered to the bone lesions. The other extrapolated treatments would have delivered lower lesion absorbed doses, ranging from –34% for ^153^Sm-EDTMP to –85% for ^166^Ho-DOTMP.

**Table 3.** Delivered (^186^Re-HEDP) and extrapolated median, minimum and maximum absorbed doses for the patient population and absorbed doses that cover 50% of the total disease volume of the population (D50)

Radiopharmaceutical	Median [min–max] absorbed dose (Gy)	Diff. from ^186^Re-HEDP (%)	D50 (Gy)
^32^P-Na_3_PO_4_	10.4 [2.3–55.4]	–41	11.5
^89^Sr-dichloride	21.8 [4.5–117.6]	25	23.1
^153^Sm-EDTMP	12.2 [3.3–57.7]	–32	13.0
^166^Ho-DOTMP	2.7 [0.6–11.8]	–85	3.7
^177^Lu-EDTMP	14.0 [3.7–72.0]	–20	14.8
^186^Re-HEDP	17.7 [3.9–87.5]	–	18.4
^188^Re-HEDP	6.3 [1.5–27.1]	–64	7.6

**Figure 3.**
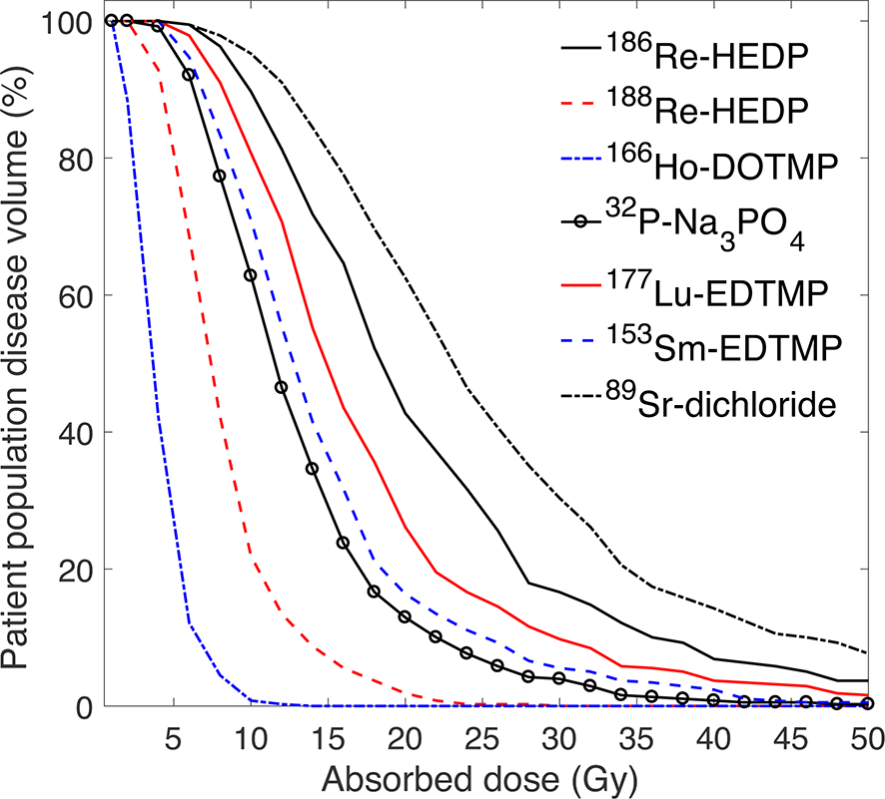
Cumulative absorbed dose volume histograms for the delivered and extrapolated treatment plans.

The absorbed dose profiles for the patient population calculated for each individual treatment were converted into cumulative dose-volume histograms, shown in Figure 3. The absorbed dose that irradiates 50% of the patient population disease volume ranged from 3.7 to 23.1 Gy for ^166^Ho-DOTMP and ^89^Sr-dichloride, respectively.

## Discussion

This study presents the methodology to calculate the absorbed dose that could be delivered for a given treatment using patient-specific sequential imaging of a delivered radiopharmaceutical. Its application to a cohort of patients with bone metastases showed that a wide range of absorbed doses can potentially be delivered to the same patient cohort from typical empirically determined activities prescribed in a range of MRT treatments in patients with mCRPC.

Radiopharmaceuticals targeting bone metastases are incorporated into bone by different mechanisms. ^89^Sr or ^223^Ra belong to the alkaline earth metals in the second group of the periodic table and have similar chemical properties and interactions to those of calcium and are, therefore, directly incorporated into the bone matrix. Other radionuclides are chelated to phosphates, such as ^153^Sm-EDTMP, ^1^^66^Ho-DOTMP, and ^177^Lu-EDTMP, ^186^Re-HEDP and ^188^Re-HEDP which are incorporated into the hydroxyapatite. These bone-seeking radiopharmaceuticals can also be classified as volume-seekers, such as earth alkaline radionuclides with long half-lives that are initially deposited in the bone surface and slowly migrate through the bone mineral by chemical exchanges, and surface-seekers such as the chelated radionuclides. Radiolabelled PSMA-binding agents are a new class of treatments for mCRPC that target PSMA-expression of prostate cancer cells and, therefore, can deliver high radiation doses to the primary tumour, lymph node and bone lesions. All these radionuclides also emit *γ*-rays or bremsstrahlung radiation, which allows imaging of the biokinetics and uptake distribution with a *γ*-camera. Presently, dosimetry is not used to guide therapy and the prescribed activity is either fixed or adjusted by patient body weight.^35^ This is likely due to the challenges associated with skeletal dosimetry, such as the variability of intraosseous trabecular distributions, the dynamic behaviour of bone marrow and the non-uniform distribution of uptake of bone-seeking radionuclides. Therefore, absolute absorbed doses delivered to the bone lesions cannot be accurately calculated, mainly due to the limited image resolution and the highly heterogeneous uptake of the radiopharmaceutical. Nonetheless, relative dosimetry can still provide valuable information on treatment efficacy when correlated with measures of treatment response and patient outcome.^13^

This study highlighted the differences in lesion absorbed doses that could be delivered to the same cohort of patients with mCRPC, if they were treated with other typical MRT administrations. Only ^89^Sr-dichloride would have delivered higher lesions (25%) doses than ^186^Re-HEDP. ^32^P-Na_3_PO_4_, ^153^Sm-EDTMP, ^166^Ho-DOTMP, ^177^Lu-EDTMP and ^188^Re-HEDP would have delivered 41, 32, 85, 20 and 64% lower absorbed doses for the typical administered activities, respectively.

This study has some limitations. An assumption of uniform uptake distribution of the radiopharmaceuticals within the bone lesions was made due to the limited spatial resolution of present clinical imaging systems. This limitation on the spatial resolution justifies the assumption of uniform uptake in the bone lesion dosimetry studies performed to date.^4, 5, 11–13, 18, 28, 36–39^ A previous study has shown that the uniformity assumption can underestimate the absorbed dose delivered to bone metastases by a factor of up to 1.85 for lesions with a higher density.^40^ The implications of this study are, however, of limited use in clinical practice, as it was based on simulations of absorbed dose distributions in a small number of biopsies from patients with mCRPC treated with ^186^Re-HEDP. Peak lesion absorbed doses were calculated to reduce the impact of partial volume effects due to the different spatial resolution of the various radionuclides. The methodology could be extended to include a point–spread function to correct the images to for the spatial resolution of the chosen radionuclide. Data on biological half-lives and bone uptake for the different radiopharmaceuticals are presently limited and the methodology employed in their calculation is often not reported in the literature. Further image quantification studies with standardized protocols traceable to primary standards of activity^41^ are needed to determine the uptake and biological retention of radiopharmaceuticals. This would in turn improve the accuracy of the extrapolations presented in this study. The ultimate significance of the range of absorbeddoses that the patient cohort studied would have received cannot be established. The efficacy of these treatments is likely dependant on the microscopic heterogeneous absorbed dose delivered to the tumorous tissue of the different radiopharmaceuticals and their associated toxicity profile. Therefore, a treatment that delivers a lower macroscopic mean lesion absorbed dose does not necessarily mean that it would be a more successful treatment. Correlations with treatment response and patient outcome are ultimately needed to understand the targeting mechanisms of the different radiopharmaceuticals available.

The methodology presented here can be used to calculate absorbed doses delivered to any target by extrapolation of the corresponding time–activity curve and could potentially be used for extrapolating bone marrow or whole body absorbed doses. This would allow personalized treatment planning by maximizing the administered activity that would deliver an optimal therapeutic absorbed dose whilst limiting toxicity to the bone marrow. A range of MRT treatments could be planned using diagnostic imaging for different therapeutic radiopharmaceuticals and a range of scan times, administered activities, uptakes and biological retentions functions. This would provide a range of treatment options to establish the optimal treatment. This methodology could be of particular importance for established and newly emerging theragnostic radiopharmaceuticals^4, 42, 43^ and for repeated treatments like ^223^Ra-dichloride, where biodistribution and pharmacokinetics have been shown to be largely consistent between administrations.^44^

## Conclusion

The methodology to extrapolate the absorbed dose that would be delivered by any radiopharmaceutical using patient-specific imaging was presented and applied to a range of MRT treatments in patients with mCRPC. For the same patient cohort, a range of lesion absorbed doses was demonstrated for typical administrations protocols, whilst the impact of various assumptions was shown. This method has the potential to be used for personalized treatment planning, in particular for emerging theragnostic radiopharmaceuticals.
